# Earth and Mind Culture

**Published:** 1854-07

**Authors:** 


### EARTH AND MIND CULTURE.

Among our monthly exchanges, we look with especial interest for the Horticultural Review and Botanical Gazette, of Cincinnati, and the Horticulturist and Journal of Rural Art and Rural Taste, published at Rochester, New York. These publications, of fifty octavo pages a month, are furnished at the very low rate of two dollars a year, to clubs of five persons and to clergymen. It would be among the grandest consummations, if these and similar publications could be made to take the place of the wishy-washy love-sick monthlies, which count their circulation by scores of thousands, polluting the hearts of our sons and our daughters, tainting their memories, corrupting their imaginations, falsifying their ideas of natural life, planting and nourishing impracticable and vain ambitions, vitiating the morals, wasting the time, perverting the affections and sympathies of our nature, and undermining the health. These are the legitimate, the necessary, the universal tendencies of novel reading, more especially of the kind found in the fashion-plate monthlies established five or six or more years ago; and yet these monthlies are regularly noticed and whitewashed by religious newspapers, with here and there an independent exception, as the Louisville Presbyterian Herald, The New York Observer, and other standard papers. How can a Christian parent allow such poisons to lie on his centre table by the side of the family Bible from one year's end to another! How can the gentleman and the scholar allow these publications to be handled by his children, when he must know that they give false ideas of life, as it is, and impart an exaggeration of style to the young mind which give the impress of "snob" for the remainder of life! You can tell the readers of these publications in five minutes' conversation, yes, in their first utterances—everything is "awful," "excruciating," "horrid," "splendid," "most magnificent." Each sight, or incident, or transaction, exceeds in the pleasurable or disagreeable anything they had "ever seen in their life before."—The truly refined, the truly aristocratic, the truly knowing, do not use these sweeping expletives, they know too much, their observation and experience has been too large, and their taste and judgment, and presence of mind, and reverence for truth forbid these wild exaggerations.

If, then, the established fashion-plate monthlies were banished from our houses, and publications on the cultivation of gardens, fruits, flowers, and various plants, were to take their place, and some means were employed at first to get our children interested in such things, a field of investigation would soon open to their view as beautiful and boundless as the universe, interesting, happyfying, and enchanting in every step of progress.

The cultivation of a single flower, on a surface of a foot square in a New York city back yard would, with proper guidance, give a more practical insight of botany, chemistry and geology, than is obtained in a whole course of four years' study in one of the "boarding schools" of the time, or rather "skinning schools," as applied to the parent's pocket, and "ruination schools" as applied to the pupil, as far as the habits of mind, body and health are concerned, as well as to the utilities of actual, practical life. I do not mean the fancy or amateur culture which the June Knickerbocker describes when

### MR. AND MRS. SPARROWGRASS RETIRE FROM THE CITY TO ENJOY RURAL LIFE.

"When Mrs. Sparrowgrass and I moved into the country, with our heads full of fresh butter, and cool, crisp radishes for tea; with ideas entirely lucid respecting milk, and a looseness of calculation as to the number in family it would take a good laying hen to supply with fresh eggs every morning; when Mrs. Sparrowgrass and I moved into the country, we found some preconceived notions had to be abandoned, and some departures made from the plans we had laid down in the little back-parlor in avenue G.

"One of the first achievements in the country is early rising! with the lark—with the sun—while the dew is on the grass,

'under the opening eyelids of the morn,' and so forth. Early rising! What can be done with five or six o'clock in town? What may not be done at those hours in the country? With the hoe, the rake, the dibble, the watering-pot? To plant, prune, drill, transplant, graft, train and sprinkle! Mrs. S. and I agreed to rise *early* in the country—

> "'Richard and Robin were two pretty men,
> They laid in the bed till the clock struck ten:
> Up jumped Richard and looked at the sky:
> O Brother Robin the sun's very high!'

Early rising in the country is not an instinct; it is a sentiment, and must be cultivated.

"A friend recommended me to send to the south side of Long Island for some very prolific potatoes—the real hippopotamus breed. Down went my man, and what with expenses of horse-hire, tavern bills, toll-gates, and breaking a wagon, the hippopotami cost as much as pine-apples. They were fine potatoes though, with comely features, and large, languishing eyes, that promised increase of family without delay. As I worked my own garden, (for which I hired a landscape gardener at two dollars per day to give me instructions,) I concluded that the object of my first experience in early rising should be the planting of the hippopotamuses. I accordingly rose next morning at five, and it rained! The next, and it rained! It rained for two weeks! We had splendid potatoes every day for dinner. 'My dear,' said I to Mrs. Sparrowgrass, 'where did you get these fine pototoes?' 'Why,' said she, innocently, 'out of that basket from Long Island!' The last of the hippotamuses were before me, peeled, and boiled, and mashed, and baked, with a nice thin brown crust on the top.

"I was more successful afterward. I did get some fine seed-potatoes in the ground. But something was the matter: at the end of the season I did not get as many out as I put in.

"Mrs. Sparrowgrass, who is a notable house-wife, said to me one day, 'Now, my dear. we shall soon have plenty of eggs, for I have been buying a lot of young chickens.' There they are, each one with as many feathers as a grasshopper, and a chirp not louder. Of course, we looked forward with pleasant hopes to the period when the first cackle should announce the milkwhite egg, warmly deposited in the hay which we had provided bountifully. They grew finely, and one day I ventured to remark that our hens had remarkably large combs, to which Mrs. S. replied, 'Yes, indeed, she had observed that; but, if I wanted to have a real treat, I ought to get up early in the morning and hear them crow.' 'Crow!' said I, faintly, 'our hens crowing! Then, by 'the cock that crowed in the morn, to wake the priest all shaven and shorn,' we might as well give up all hopes of having eggs,' said I, 'for, as sure as you live, Mrs. S., our hens are all roosters!' And so they were roosters! that grew up and fought with the neighbors' chickens, until there was not a whole pair of eyes on either side of the fence.

"A *dog* is a good thing to have in the country. I have one which I raised from a pup. He is a good, stout fellow, and a hearty barker and feeder. The man of whom I bought him said he was thorough-bred, but he begins to have a mongrel look about him. He is a good watch-dog though, for the moment he sees any suspicious-looking person about the premises, he comes right into the kitchen and gets behind the stove. First we kept him in the house, and he scratched all night to get out. Then we turned him out, and he scratched all night to get in. Then we tied him up at the back of the garden, and he howled so that our neighbor shot at him twice before daybreak. Finally, we gave him away, and he came back; and now he is just recovering from a fit in which he has torn up the patch that had been sown for our spring radishes.

"A good strong gate is a necessary article for your garden. A good, strong, heavy gate, with a dislocated hinge, so that it will neither open nor shut. Such an one had I last year. The grounds before my fence are in common, and all the neighbors' cows pasture there. I remarked to Mrs. S., as we stood at the window in June last, how placid and picturesque the cattle looked, as they strolled about, cropping the green herbage. Next morning I found the innocent creatures in my garden. They had not left a green thing in it. The corn in the milk, the beans on the poles, the young cabbages, the tender lettuce, even the thriving shoots on my young fruit trees had vanished. And there they were, looking quietly on the ruin they had made. Our wath-dog, too was foregathering with them. It was too much, so I got a large stick and drove them all out, except a young heifer, whom I chased all over the flower-beds, breaking down my trellises, my woodbines and sweet-briars, my roses and petunias, until I cornered her in the hot-bed. I had to call assistance to extricate her from the sashes, and her owner sued me for damages and recovered. I believe I shall move in town."

The above is not the earth culture we are inculcating, but the practical cultivation of a few square feet of ground which may be had in any city back yard, taking some Horticultural monthly, as a guide, with an elementary work on Botany, Chemistry, and Geology; thus a single article on the moss-rose, or morning-glory, or strawberry, or holly hock, or any familiar plant or flower, would give abundant study for a month, and all the while delighting and expanding the mind.

If all the fashion-plate monthlies in the land could be made into one vast hecatomb of fire, or, if the paper could be remade and used for Horticultural Magazines, and every family were to subscribe, and pay for, and practically use them, and no Bremer, or D'Israeli, or Dickens, or Sue, or Dumas, or George Sand, or Thackeray, was allowed a place in the family library, this would be a far more virtuous, and refined, and practical, and happy world than it is: our sons would be more like men, for their minds would be agreeably exercised on subjects which naturally elevate: our daughters would make better wives, for instead of having their imaginations, and sensibilities and sympathies expended in a wrong direction, instead of originating, cherishing, and feeding expectations, never deserved, never to be realized, and really, never truly desirable, their mental, moral, and physical powers would be usefully employed on the topics discussed in the monthlies above named. There is something ennobling, something pacifying in the cultivation of the soil. It eventually makes a man independent, self-reliant. The earth gives her retnrns without demanding of her customers every variety of bowings, and scrapings, and servilities, and meanness, and misrepresentations, and stretchings, and downright cheateries, so general now among all trades and traffics. An active man in business, and a money-making fellow, said in my hearing not long ago, "I hate my business, I loathe and abhor it. I must lie and cheat, or fail, for my rivals live by it." This was said by a young man who had one of the best mothers that ever drew the breath of life.—How a man's pen will run away!

It is now writing of mercantile morals in a notice of a publication on gardening. I hope the reader has been wide enough awake to see the connection—although it is an idea which I do not recollect to have seen—that the cultivation of the soil naturally dignifies, elevates, and ennobles the character, because the earth yields her increase without demanding the flatteries and falsities of trade, while its tillers have a right, by means of the promise, to expect and demand an ample return from well-directed labor; our great mother gives full measure, pressed down and running over; no twelve cents for a shilling, no thirty-four inches for a yard, no fifty-six grains for a dram, no fourteen ounces, avordupois for a pound, no watered milk, no sanded sugar, no chicoried coffee, no drugged wines, no manufactured nutmegs or wooden seeds.

Let any young woman read the article on the chemistry of organic cells, in the Cincinnati Horticultural Review, let her read it with care and see if she does not rise from the perusal of a two-paged article with a feeling of elevation of mind which arises from the consciousness of increased knowledge, which no novel can ever similate, and which freshens, and expands, and deepens for life-long observation; while the stories and the tales, like the exhilarations from brandy or opium, fade away forever, leaving only the poison, and the sting behind: the poison of deliberate harm done to the body, the sting of time misspent, never to be regained.

